# Supplementary material for: The microbiome biomarkers of pregnant women’s vaginal area predict preterm prelabor rupture in Western China
Source: Front Cell Infect Microbiol. 2024 Oct 31;14:1471027. doi: 10.3389/fcimb.2024.1471027 (PMC11560878; doi:10.3389/fcimb.2024.1471027)
Supplement: Supplementary file 1 [file DataSheet1.zip › compare_1/Community/Pieplot/class/sample/P35.class.pieplot.pdf]

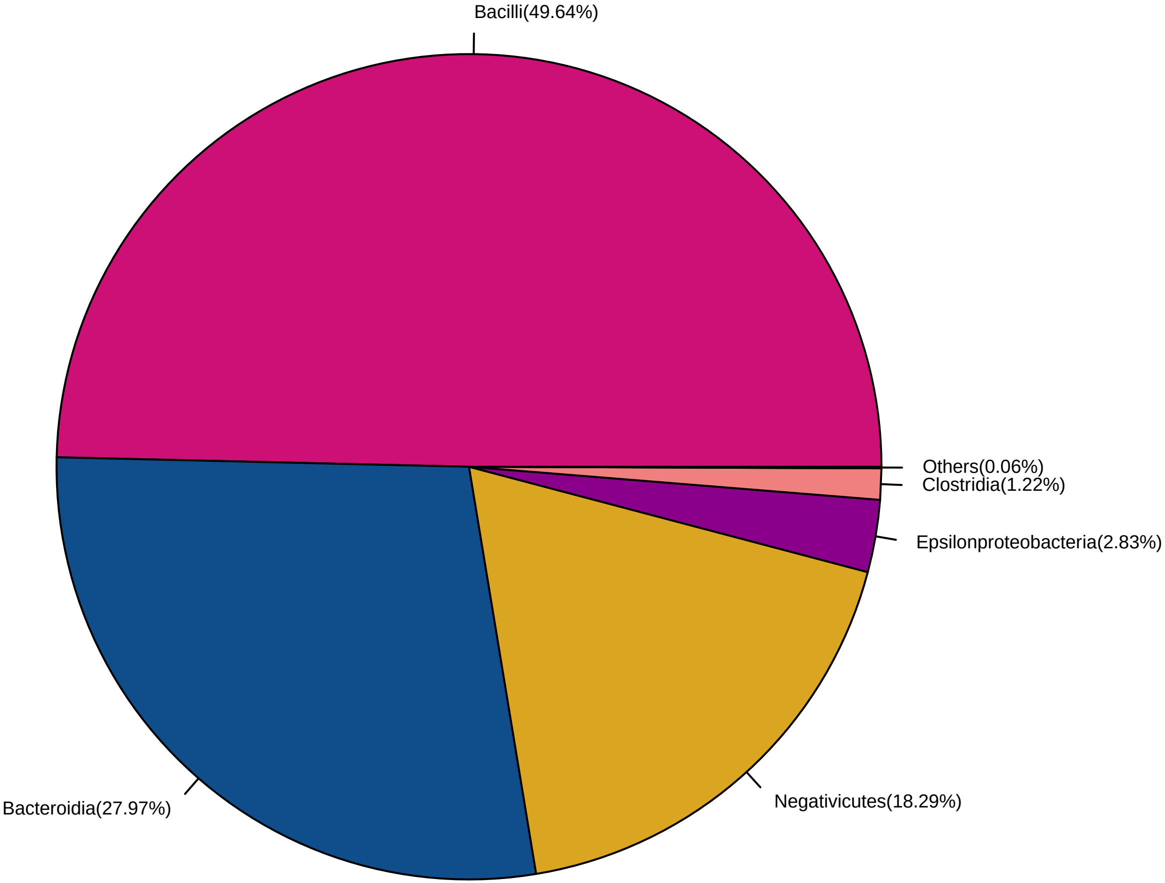

Bacilli(49.64%)
Others(0.06%)
Clostridia(1.22%)
Epsilonproteobacteria(2.83%)
Negativicutes(18.29%)
Bacteroidia(27.97%)
